# Zingerone Targets LKB1/AMPK to Block FcεRI-Dependent Mast Cell Degranulation and Anaphylaxis

**DOI:** 10.3390/cimb47110963

**Published:** 2025-11-19

**Authors:** Defeng Zheng, Hui Zhang, Can Mao, Jinqiang Liang, Xian Li

**Affiliations:** Key Laboratory of Tropical Biological Resources of Ministry of Education and One Health Institute, School of Pharmaceutical Sciences, Hainan University, Haikou 570228, China

**Keywords:** Zingerone, anaphylaxis, mast cell, IgE, AMPK

## Abstract

AMP-activated protein kinase (AMPK) acts as a cellular energy sensor and a central regulator of metabolism. Recent studies indicate that pharmacological AMPK activation can simultaneously ameliorate metabolic disorders (e.g., type II diabetes, obesity) and allergic diseases. Zingerone, a primary bioactive compound in ginger, demonstrates protective effects in vascular calcification, non-alcoholic fatty liver disease, and asthma via AMPK activation. This study aimed to evaluate the anti-allergic activity of Zingerone and elucidate its AMPK-dependent mechanisms. In vitro, Zingerone suppressed FcεRI-mediated phosphorylation of PLCγ1, Akt, ERK1/2, JNK, p38, and IKK, while reducing β-hexosaminidase release, eicosanoid (LTC_4_/PGD_2_) generation, pro-inflammatory cytokine (TNF-α/IL-6) secretion, and Ca^2+^ influx through LKB1/AMPK activation. In vivo, Zingerone (25–50 mg/kg, oral) attenuated passive cutaneous anaphylaxis (reduced Evans blue extravasation) and systemic anaphylaxis (inhibited histamine/LTC_4_/PGD_2_ release). These findings demonstrate that Zingerone inhibits FcεRI-dependent mast cell activation and anaphylaxis via the LKB1/AMPK pathway, highlighting its therapeutic potential for mast cell-mediated allergic diseases.

## 1. Introduction

Systemic anaphylaxis is an acute, IgE-mediated lethal reaction triggered by re-exposure to allergens [1]. The high-affinity FcεRI receptor, predominantly expressed on mast cells, is pivotal in allergic pathogenesis [2]. Allergen binding to IgE-FcεRI complexes activates downstream signaling (e.g., Lyn/Fyn/Syk kinases), leading to PLCγ, MAPK, Akt, and NF-κB pathway activation. This cascade culminates in mast cell degranulation and release of histamine, proteases, eicosanoids (PGD_2_/LTC_4_), and cytokines, driving allergic manifestations [3,4,5]. Aggregation of IgE bound FcεRI by antigen (Ag) leads to activation of proximal Src kinases (Lyn and Fyn) and Syk, thereby activating multiple pathways including phospholipase Cγ (PLCγ), mitogen-activated protein kinase (MAPK), Akt and NF-κB, resulting in the release of preformed mediators and cytokines [6,7,8]. Till now, allergy therapy has focused on histamine receptor antagonists, inhibition of free histamine and production of inflammatory factors such as PGD_2_ and LTC_4_. Most drugs used for anti-allergic are immunosuppressants and antihistamines, which temporarily relieve symptoms but can be accompanied by various side effects. Therefore, under the existing treatment concept, there is an urgent need for therapeutic agents with new concepts.

AMPK, the “metabolic master switch,” senses cellular energy status and regulates processes including lipid metabolism, insulin sensitivity, and inflammation [9,10,11,12]. Recent studies have shown that artificial activation of the sensor protein AMPK, used to maintain the energy integrity in the body, can not only treat metabolic diseases such as type II diabetes, obesity and fatty liver, but also inhibit allergic and inflammatory reactions [13,14,15,16]. At present, in order to control disease and progression, safe and healthy treatment is needed. Related to this, medicinal plants and their components play an important role in disease management by regulating biological activities. Many medicinal plant components have antioxidant, anti-inflammatory, anti-diabetic and anti-tumor properties, which are of great significance to disease treatment.

Zingerone, a bioactive compound derived from ginger, exhibits a broad spectrum of pharmacological properties including antioxidant, anti-inflammatory, and AMPK-activating activities [17,18]. Previous studies have demonstrated that Zingerone can activate AMPK in different pathological contexts; for example, it was shown to ameliorate non-alcoholic fatty liver disease (NAFLD) in rats through AMPK pathway activation and to protect against vascular calcification via AMPK-dependent signaling. Separately, evidence also supports the anti-allergic potential of Zingerone, as shown in a murine asthma model where it alleviated airway inflammation [19,20,21,22]. However, despite these individual lines of evidence, it remained unclear whether Zingerone’s anti-allergic effect is mechanistically linked to AMPK activation specifically in the context of FcεRI-mediated mast cell activation, a key pathway in allergic reactions. The role of the upstream kinase LKB1 and the functional impact of the LKB1/AMPK axis on mast cell degranulation and anaphylaxis had not been explored. This knowledge gap led us to hypothesize that Zingerone suppresses mast cell-driven allergic responses primarily via activation of the LKB1/AMPK signaling pathway. In this study, we therefore investigated whether Zingerone inhibits FcεRI-dependent mast cell activation and anaphylaxis by activating the LKB1/AMPK axis, thereby providing a mechanistic basis for its anti-allergic activity.

## 2. Materials and Methods

### 2.1. Reagents

Antibodies against phosphorylated/total LKB1 (Ser428), AMPKα (Thr172), ACC (Ser79), PLCγ1 (Tyr783), Akt (Ser473), p38, ERK1/2, JNK, IKKα/β and GAPDH were sourced from Cell Signaling Technology (Danvers, MA, USA). Anti-DNP IgE and DNP-HSA were obtained from Sigma-Aldrich (St. Louis, MO, USA), Zingerone, and AICAR were purchased from MedChemExpress (Monmouth Junction, NJ, USA) were used. Stock solutions: AICAR (400 mM in H_2_O) and Zingerone (40 mM in DMSO).

### 2.2. Animals

BALB/cJ and ICR mice were supplied by SpePharm Biotechnology (Beijing, China) were housed under specific pathogen-free conditions (22 °C, 40–70% humidity, 12 h light/dark cycle). All procedures followed Hainan University IACUC guidelines.

### 2.3. Culture and Activation of Mouse Bone Marrow-Derived Mast Cells and RBL-2H3 Cells

Bone marrow-derived mast cells (BMMCs) from male BALB/cJ mice [23] and RBL-2H3 cells were cultured as described. Cells (1 × 10^6^/mL) were sensitized with anti-DNP IgE (500 ng/mL, overnight) and stimulated with DNP-HSA (100 ng/mL) [14]. Zingerone or AICAR was added 2 h or 5 h pre-stimulation. Degranulation (β-hexosaminidase release), LTC_4_/PGD_2_ and cytokines (ELISA kits from R&D Systems, Minneapolis, MN, USA) were quantified.

### 2.4. CCK-8 Assay

BMMCs and RBL-2H3 cells in logarithmic growth phase were harvested and resuspended to a density of 5 × 10^4^ cells/mL. The suspension was gently mixed to ensure homogeneity, then seeded into 96-well plates (100 μL/well). Control groups received 0.5 μL DMSO per well, while experimental groups were treated with 0.5 μL zingerone at final concentrations of 1, 5, 10, 20, or 50 μM (ascending gradient). Each concentration was tested in triplicate wells. After 8 h of incubation (37 °C, 5% CO_2_), 10 μL CCK-8 solution was added to each well, followed by additional incubation for 1 h under identical conditions. Absorbance was measured at 450 nm using a microplate reader, with data recorded for subsequent analysis.

### 2.5. Measurement of Intracellular Ca^2+^ Level

Intracellular calcium levels were quantified using the FluoForte Calcium Assay Kit (Enzo Life Sciences, Ann Arbor, MI, USA), following a protocol consistent with our previous work [13]. Briefly, IgE-sensitized bone marrow-derived mast cells (BMMCs) were incubated with FluoForte Dye-Loading Solution for 1 h at room temperature. After removing excess dye by washing with HBSS, the cells (5 × 10^4^) were plated into 96-well microplates. Subsequently, they were pretreated with the indicated concentrations of zingerone for 1 h or AICAR for 5 h prior to stimulation with DNP-HSA. Fluorescence was measured using microplate reader at excitation and emission wavelengths of 485 nm and 520 nm, respectively.

### 2.6. Immunoblotting

Immunoblotting analysis was performed the same protocol as described previously [23]. IgE-sensitized mast cells were stimulated with antigen (Ag) for specified durations in the presence or absence of Zingerone or AICAR. Whole-cell lysates equivalent to 1 × 10^6^ cells were resolved on 8% SDS-PAGE gels under reducing conditions and transferred to PVDF membranes. Immunoblotting was performed using phospho-specific antibodies against LKB1 (Ser428), AMPKα (Thr172), ACC (Ser79), PLCγ1 (Tyr783), Akt (Ser473), p38, ERK1/2, JNK, and IKKα/β, alongside total protein antibodies against corresponding targets and GAPDH as a loading control. Membranes were incubated with HRP-conjugated goat anti-rabbit IgG secondary antibody (1:3000 dilution) for 1 h, followed by protein visualization using enhanced chemiluminescence (ECL, Beyotime Biotechnology, Shanghai, China).

### 2.7. Immunofluorescence Assay

The subcellular localization and activation of AMPK were examined by immunofluorescence assay followed by confocal microscopy. RBL-2H3 cells were cultured on glass coverslips, sensitized with anti-DNP IgE, and pretreated with or without Zingerone (10–20 μM) for 1–2 h at 37 °C before stimulation with DNP-HSA (100 ng/mL) for 15 min. Cells were fixed with 4% paraformaldehyde, permeabilized with 0.1% Triton X-100, and blocked with 5% bovine serum albumin (BSA). Samples were then incubated overnight at 4 °C with a phospho-specific AMPK (Thr172) primary antibody. After washing, a CY3-conjugated secondary antibody (red fluorescence) was applied, and nuclei were stained with DAPI (blue). Coverslips were mounted using an anti-fade medium to preserve fluorescence signals.

Image Acquisition and Processing:

Confocal images were acquired at high resolution with optimized laser power, gain, and offset settings for both CY3 (AMPK) and DAPI (nuclei) channels to ensure clear separation between nuclear and cytoplasmic compartments while avoiding signal saturation. Scale bars were included in all images, and merged images accurately represented signal co-localization.

Quantification of Fluorescence Intensity:

Fluorescence intensity was quantified as the mean fluorescence intensity (MFI) of the phosphorylated AMPK signal (CY3) specifically within the cytoplasmic region of individual cells. The cytoplasmic area was defined by subtracting the DAPI-defined nuclear region from the whole-cell area. This approach ensured that measurements reflected AMPK activation in its functional cytoplasmic context. Image analysis was performed using ImageJ software (Version 1.53t, National Institutes of Health, Bethesda, MD, USA; accessed on 20 May 2025) with appropriate thresholding to exclude background signal.

### 2.8. Transfection of siRNA

Transfection experiments were carried out as described previously [13]. SiRNA transfection experiments utilized ON-TARGETplus SMARTpool reagents targeting rat LKB1 and AMPKα2 (Dharmacon, Lafayette, CO, USA), which employ a pooled design of four distinct siRNAs per target to ensure >75% gene silencing efficiency. Non-specific siRNA (Santa Cruz Biotechnology, Inc., Dallas, TX, USA) served as the negative control to account for off-target effects.

### 2.9. IgE-Mediated Local and Systemic Anaphylaxis in Mice

Passive cutaneous anaphylaxis (PCA) was carried out by the same protocol as described previously [13]. Passive cutaneous anaphylaxis (PCA) was induced in 6-week-old male mice by intradermal injection of 80 ng anti-DNP IgE into one ear. After 24 h, mice were orally administered Zingerone (25 or 50 mg/kg) or fexofenadine HCl (50 mg/kg), followed 1 h later by intravenous challenge with 60 μg DNP-HSA containing Evans blue (*n* = 10/group). Evans blue extravasation was quantified after 1 h by extracting ear tissue in formamide (63 °C overnight) and measuring absorbance at 630 nm. For passive systemic anaphylaxis (PSA) [14], mice received 2 μg DNP IgE intravenously, and after 24 h, were orally treated with Zingerone or fexofenadine HCl for 1 h before intravenous challenge with 4 mg DNP-HSA in 100 μL saline. Blood was collected via cardiac puncture 5 min post-challenge, and serum histamine, LTC_4_, and PGD_2_ levels were quantified using commercial immunoassay kits (Novus Biologicals, LLC, Centennial, CO, USA). Rationale for positive control selection: Fexofenadine HCl, a potent H1-antihistamine, was selected as the positive control based on its clinical relevance and alignment with our study objectives. As histamine represents one of the earliest and most critical mediators released during IgE-mediated anaphylaxis, and fexofenadine is a first-line clinical treatment for type I hypersensitivity reactions, it provides a clinically meaningful reference for evaluating Zingerone’s anti-allergic efficacy. While mast cell stabilizers like Cromolyn sodium represent an alternative mechanistic approach, fexofenadine’s well-established efficacy in blocking histamine-mediated responses and its consistent in vivo performance better aligned with the endpoints measured in our models.

### 2.10. Statistic Analysis

Statistical analyses utilized GraphPad Prism 9.0, employing unpaired t-tests for two-group comparisons and one-way ANOVA with appropriate post hoc tests for multiple-group comparisons. All quantitative data are expressed as means ± SEM, with statistical significance defined at *p* < 0.05.

## 3. Results

### 3.1. Assessment of Zingerone Cytotoxicity by CCK-8 Assay

**Figure 1:**
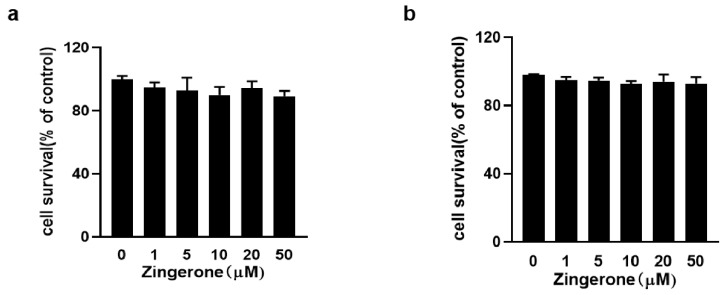
Zingerone demonstrated no cytotoxicity toward BMMCs (**a**) and RBL-2H3 cells (**b**) after 8 h of incubation across concentrations of 1–50 μM, as quantified by CCK-8 assays.

The cytotoxicity of Zingerone on mast cells was evaluated using the CCK-8 assay. Initial assessment after 8 h of incubation demonstrated that Zingerone exhibited no significant cytotoxicity across a concentration range of 1–50 μM, with cell viability sustained above 95% relative to untreated controls even at the highest concentration of 50 μM (Figure 1a,b). This result confirmed the high cytocompatibility of Zingerone at the concentrations used for subsequent short-term in vitro mechanistic studies. To address the possibility that the observed inhibitory effects might be attributable to delayed toxicity or cellular stress arising from longer exposure periods, a supplementary experiment was conducted. In this follow-up assay, both BMMCs and RBL-2H3 cells were treated with the highest tested concentration of Zingerone (50 μM) for an extended duration of 24 h. The results demonstrated that even after this prolonged exposure, cell viability in the Zingerone-treated groups remained high (>90%) and showed no statistically significant difference compared to the solvent control groups. This new dataset, included as Supplementary Figure S1, robustly confirms that the inhibitory effects on mast cell activation reported in this study are indeed due to the specific pharmacological activity of Zingerone, rather than non-specific cytotoxicity or cellular stress.

### 3.2. Zingerone Suppresses IgE/Ag-Induced Mast Cell Activation Through Potentiation of the LKB1-AMPK Signaling Axis

**Figure 2:**
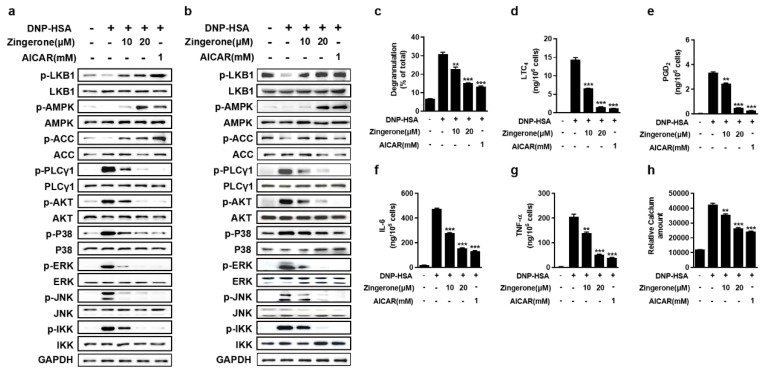
Zingerone suppressed IgE/Ag-mediated mast cell activation in BMMCs. (**a**,**b**) IgE-sensitized BMMCs were pretreated with graded Zingerone concentrations (10, 20 μM, 2 h) followed by DNP-HSA (Ag) stimulation (15 min–8 h). Immunoblotting of cell lysates demonstrated dose-dependent inhibition of FcεRI-proximal signaling phosphoproteins. (**c**–**h**) Zingerone pretreatment significantly inhibited various Ag-induced effector functions in mast cells. Specifically, it suppressed degranulation (β-hexosaminidase release at 15 min), the synthesis of lipid mediators (LTC_4_ at 15 min and PGD_2_ at 8 h), the secretion of pro-inflammatory cytokines (TNF-α and IL-6 at 6 h), and calcium mobilization (intracellular Ca^2+^ influx at 5 min), as quantified in panels (**c**–**h**), respectively. The immunoblot data (**a**,**b**) is a representative of three independent experiments, and the values (**c**–**h**) indicate the means ± S.E.M. from three independent experiments (** p < 0.01 and *** *p* < 0.001 vs. DNP-HSA alone).

Zingerone has been reported to activate the AMPK pathway in different diseases [18,24,25]. Zingerone concentration-dependently potentiated LKB1-mediated AMPKα-Thr172 phosphorylation in IgE-sensitized BMMCs, concurrently enhancing downstream acetyl-CoA carboxylase (ACC) phosphorylation. This activation suppressed FcεRI proximal signaling in both BMMCs and RBL-2H3 cells, evidenced by significant inhibition of phosphorylation in key mediators including PLCγ1, Akt, MAPKs (p38/ERK/JNK), and IKK (Figure 2a,b). Zingerone significantly suppressed multiple IgE/Ag-triggered effector responses in BMMCs, including β-hexosaminidase release, lipid mediator synthesis (LTC_4_ and PGD_2_), pro-inflammatory cytokine secretion (TNF-α and IL-6), and intracellular Ca^2+^ mobilization (Figure 2c–h). These results demonstrate that Zingerone inhibits mast cell activation by targeting the LKB1-AMPK axis to disrupt FcεRI signaling cascades and downstream inflammatory responses.

### 3.3. Zingerone Regulates the Subcellular Localization of AMPK

**Figure 3:**
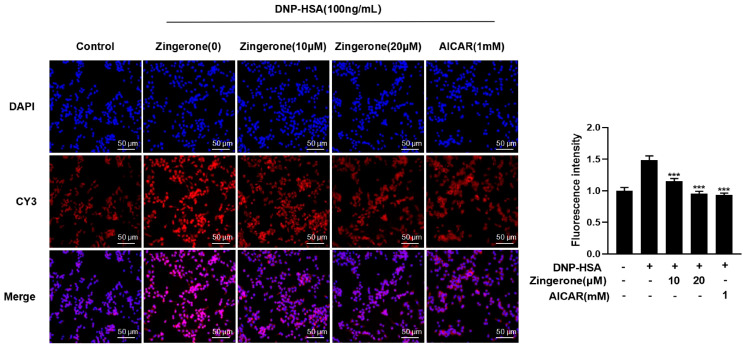
Zingerone improves AMPK enucleation by mast cell activation. The data were expressed as mean ± standard error (SEM), (*** *p* < 0.001 vs. DNP-HSA alone).

As AMPK shuttles between cytoplasm and nucleus, and only cytoplasmic AMPK can block FcεRI conduction in cells [13]. As illustrated in Figure 3, mast cells underwent specific modeling and drug treatments. Subsequently, immunofluorescence staining was performed to visualize AMPK (using red-fluorescent CY3 conjugate) and nuclei (using blue-fluorescent DAPI). Confocal laser scanning microscopy revealed the distribution of both fluorescent signals. The red CY3 signal localized AMPK protein within mast cells, while the blue DAPI signal marked the nuclei. Co-localization analysis of these images confirmed mast cell positioning and delineated the subcellular distribution of AMPK between the nucleus and cytoplasm. Figure 3 demonstrates that in the control group, AMPK exhibited relatively uniform distribution within mast cells. However, upon mast cell activation (in the allergic model), nuclear AMPK fluorescence intensity significantly increased, accompanied by a decrease in cytoplasmic AMPK signal. This shift indicates nuclear translocation of AMPK migration from the cytoplasm to the nucleus following cellular activation. Treatment with zingerone markedly attenuated this translocation phenomenon. Given that AMPK exerts its primary function within the mast cell cytoplasm, these observations suggest that mast cell activation and subsequent allergic response lead to AMPK inhibition, resulting in its nuclear accumulation. Conversely, zingerone promotes AMPK phosphorylation, facilitating its redistribution back to the cytoplasm.

Collectively, these findings indicate that zingerone counteracts activation-induced AMPK nuclear translocation. This suggests zingerone’s anti-allergic effect involves enhancing cytoplasmic AMPK localization, thereby activating the AMPK signaling axis. This activation inhibits FcεRI-mediated mast cell activation.

### 3.4. Genetic Silencing of AMPK or LKB1 Abrogates Zingerone’s Suppression of IgE/Ag-Induced Mast Cell Activation

**Figure 4:**
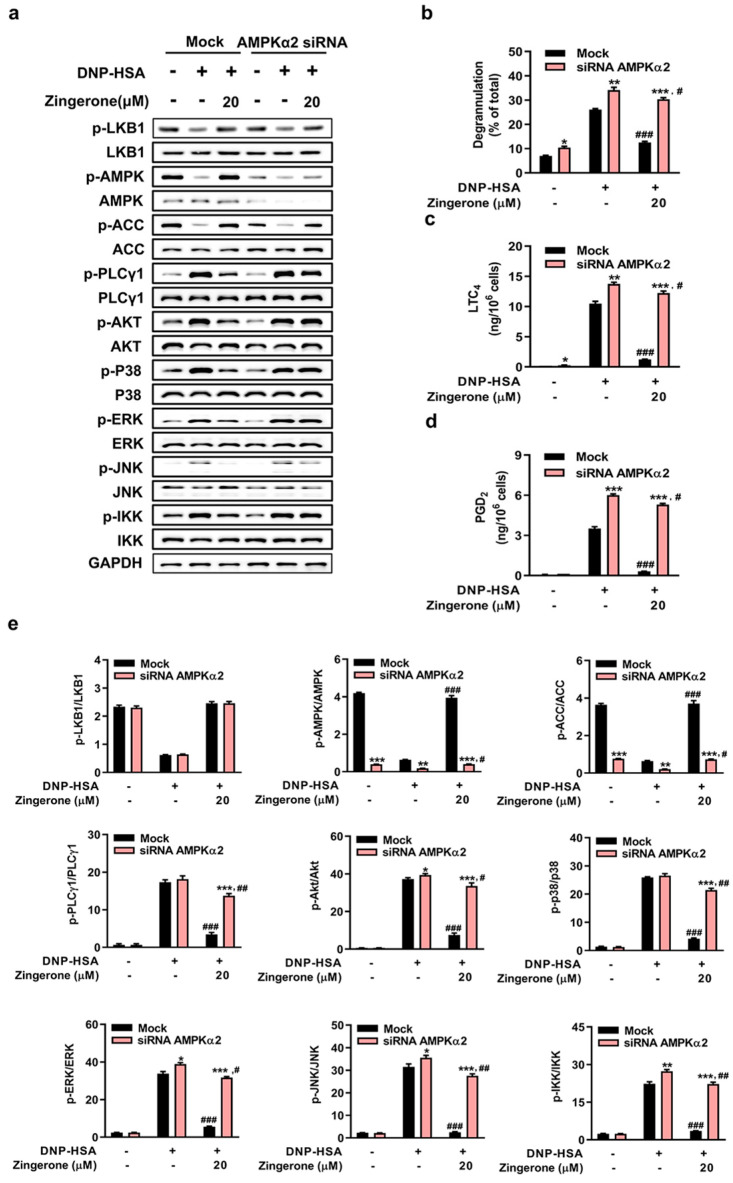
Geneticsilencing of AMPKα2 abolishes Zingerone’s anti-allergic effects in IgE/Ag-stimulated BMMCs. BMMCs transfected with AMPKα2-specific siRNA (48 h) or control siRNA (Mock) were IgE-sensitized, pretreated with 20 μM Zingerone (2 h), and stimulated with DNP-HSA (15 min). Immunoblotting revealed that Zingerone’s suppression of FcεRI-proximal signaling molecules (e.g., PLCγ1, Akt, MAPKs) was abolished in AMPKα2-silenced cells but maintained in Mock controls (**a**). AMPKα2 knockdown eliminated Zingerone’s inhibition of: Degranulation (β-hexosaminidase release) Lipid mediator synthesis (LTC_4_ and PGD_2_) release levels in siRNA-treated cells reverted to those of untreated controls (**b**–**d**). Densitometric quantification of signaling phosphoproteins in AMPKα2-knockdown BMMCs showed phosphorylated/total protein intensity ratios (**e**). Quantitative data represent mean ± SEM from three independent experiments. Significance: * *p* < 0.05, ** *p* < 0.01 and *** *p* < 0.001 vs. mock in each treatment; # *p* < 0.05, ## *p* < 0.01 and ### *p* < 0.001 vs. DNP-HSA alone in mock or knockdown group.

**Figure 5:**
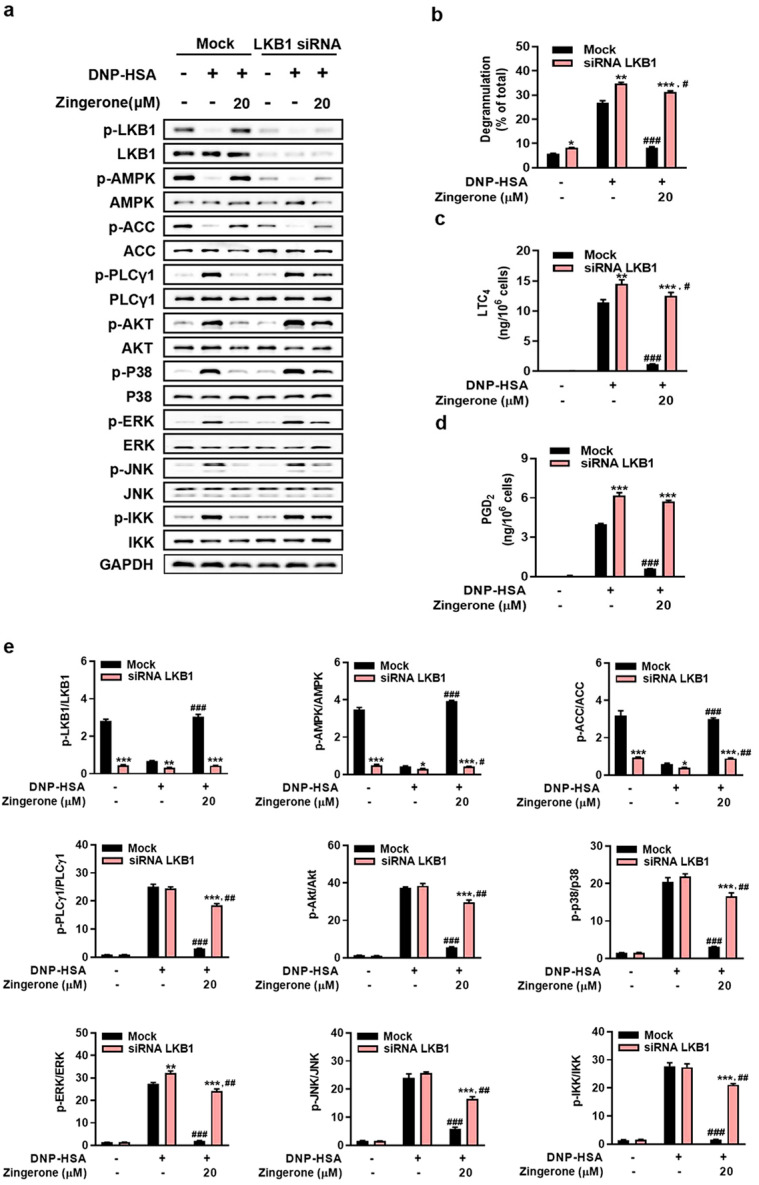
LKB1 knockdown abolishes Zingerone’s anti-allergic effects in IgE-stimulated BMMCs. BMMCs transfected with LKB1-specific siRNA (48 h) or control siRNA (Mock) were IgE-sensitized, pretreated with 20 μM Zingerone (2 h), and stimulated with DNP-HSA (15 min). Immunoblotting revealed that Zingerone’s suppression of FcεRI-proximal signaling molecules (e.g., PLCγ1, Akt, MAPKs) was abolished in LKB1-silenced cells but maintained in Mock controls (**a**), LKB1 knockdown also eliminated Zingerone’s suppression of degranulation (β-hexosaminidase release) and lipid mediator synthesis (LTC_4_ and PGD_2_) release levels (**b**–**d**), Densitometric quantification of signaling phosphoproteins in LKB1-knockdown BMMCs showed phosphorylated/total protein intensity ratios (**e**). The values indicate the means ± S.E.M. from three independent experiments with significance denoted (* *p* < 0.05, ** *p* < 0.01 and *** *p* < 0.001 vs. mock in each treatment; # *p* < 0.05, ## *p* < 0.01 and ### *p* < 0.001 vs. DNP-HSA alone in mock or knockdown group).

Genetic silencing of AMPKα2 or LKB1 in BMMCs abolished Zingerone’s suppression of FcεRI proximal signaling (phosphorylation of PLCγ1, Akt, MAPKs [p38/ERK/JNK], and IKK) and downstream effector functions (β-hexosaminidase release, LTC_4_/PGD_2_ synthesis). Specifically: AMPKα2 knockdown eliminated Zingerone-induced AMPKα2/ACC phosphorylation and restored FcεRI-triggered signaling activation and mediator release to control levels (Figure 4a–e). LKB1 knockdown prevented Zingerone-mediated LKB1/AMPK/ACC phosphorylation and abrogated its inhibition of FcεRI signaling cascades (Figure 5a–e). Critically, Zingerone enhanced LKB1 phosphorylation even in AMPKα2-silenced cells, indicating its primary target resides upstream of AMPK (likely LKB1 itself or upstream regulators), not AMPK directly.

### 3.5. Zingerone Suppresses IgE-Mediated Passive Cutaneous Anaphylaxis (PCA) in Mice

**Figure 6:**
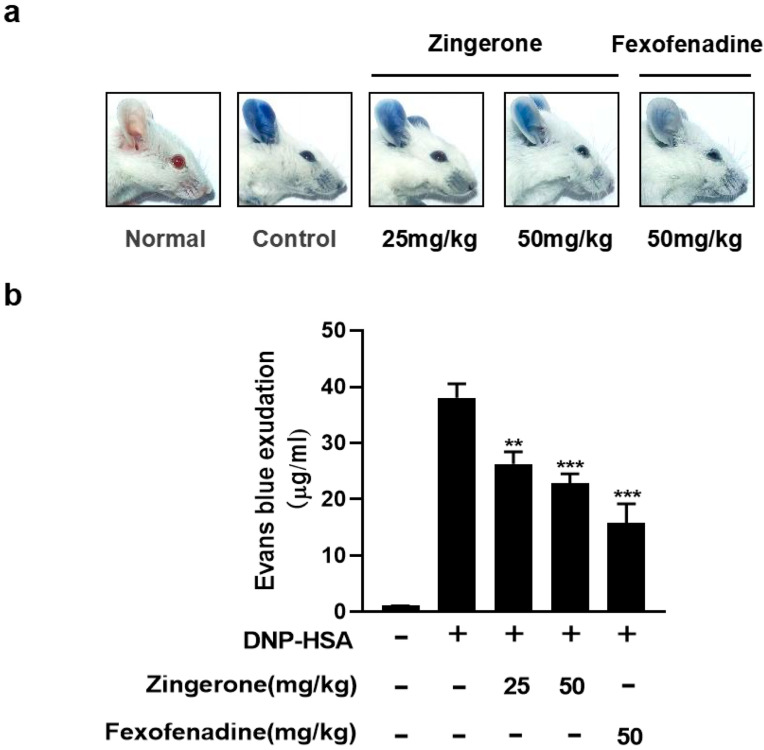
Effect of Zingerone on IgE/Ag-induced passive cutaneous anaphylaxis (PCA) reaction. Anti-DNP IgE (80 ng) was intradermally injected into mouse ears. After 24 h, mice received oral Zingerone (25 or 50 mg/kg) or fexofenadine-HCl (50 mg/kg), followed 2 h later by intravenous challenge with DNP-HSA/Evans blue (60 μg). Ear tissue dye extravasation was quantified after 1 h by formamide extraction at 63 °C overnight and quantified by absorbance at 630 nm (**a**). Dye exudation in ear tissues was quantified by Material and Methods (**b**). Data represent mean ± S.E.M. (** *p* < 0.01, *** *p* < 0.001 were compared to IgE/Ag-sensitized mice), n = 10 mice/group.

Allergic reaction is caused by crosslinking of IgE specifically bound by the allergen to FcεRI [13]. To investigate the potential effects of ginger on IgE/Ag-induced allergic reactions, ICR mice were used to evaluate the effect of Zingerone on IgE/Ag-induced passive cutaneous anaphylaxis (PCA) in vivo. Mice treated with Zingerone 25 and 50 mg/kg showed a significant dose-dependent reduction in extravasation of Evans blue at the ear tissue site, using 50 mg/kg of the H1 blocker fexofenadine hydrochloride as a control (Figure 6a,b).

### 3.6. Zingerone Inhibits IgE-Induced Passive Systemic Allergic Reaction (PSA) in Mice

**Figure 7:**
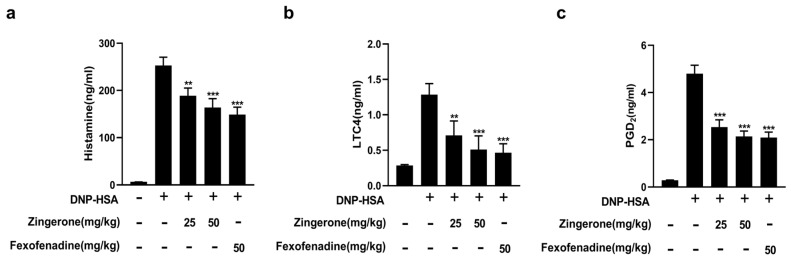
Zingerone suppresses IgE-mediated anaphylactic mediator release in mice. ICR mice intravenously injected with 2 μg IgE (vs. saline controls) were pretreated orally with Zingerone (25 or 50 mg/kg) or fexofenadine-HCl (50 mg/kg) 24 h later. After 2 h, mice were challenged intravenously with 4 mg DNP-HSA; blood was collected 5 min post-challenge. Zingerone significantly inhibited serum histamine, LTC_4_, and PGD_2_ levels (quantified by enzyme immunoassays) in a dose-dependent manner (**a**–**c**). n = 8 mice in each group. The values indicate the mean ± S.E.M. (** p < 0.01 and *** *p* <0.01 versus IgE/Ag-sensitized mice).

We evaluated the anti-allergic inflammatory effects of Zingerone using the passive systemic allergy (PSA) model. As shown, DNP-HSA was injected through the tail vein of mice at 2 h after 25, 50 mg/kg Zingerone or 50 mg/kg of fexofenadine hydrochloride 50 mg/kg. Zingerone reduced serum histamine (Figure 7a), LTC_4_ (Figure 7b), and PGD_2_ (Figure 7c) levels in a dose-dependent manner (n = 8). Fexofenadine hydrochloride with an H1 blocker of 50 mg/kg was used as a control.

## 4. Discussion

This study demonstrates that zingerone, a major bioactive compound derived from ginger, effectively suppresses FcεRI-mediated mast cell activation and anaphylactic responses in both in vitro and in vivo settings. The findings provide compelling evidence that this anti-allergic effect is primarily mediated through activation of the LKB1/AMPK signaling axis. Zingerone enhanced the phosphorylation of LKB1 and AMPK, while also significantly inhibiting downstream signaling events including PLCγ1, Akt, MAPKs (ERK, JNK, p38), and IKK. Consequently, it reduced mast cell degranulation, lipid mediator synthesis, pro-inflammatory cytokine secretion, and calcium influx. Genetic knockdown experiments confirmed that the protective effects of zingerone are dependent on the LKB1/AMPK pathway. These results highlight the potential of zingerone as a therapeutic candidate for mast cell-mediated allergic diseases via modulation of a fundamental immunometabolic signaling pathway.

### 4.1. Zingerone as a Putative LKB1 Activator and Its Downstream Signaling

A pivotal finding of this study is that zingerone enhanced LKB1 phosphorylation even under AMPKα2-knockdown conditions in mast cells. This indicates that the primary molecular target of zingerone likely resides upstream of AMPK, possibly LKB1 itself or its direct regulators. Whereas AMPK is an established negative regulator of FcεRI signaling, our data identify zingerone as a specific activator of the LKB1/AMPK axis. This mechanism distinguishes zingerone from canonical AMPK activators such as AICAR or metformin, which often influence cellular energy status (e.g., AMP/ATP ratio) or act through indirect means [26,27]. The ability of zingerone to promote LKB1 phosphorylation independent of AMPK status suggests a more direct interaction with this upstream kinase, initiating a signaling cascade that ultimately suppresses multiple pathways downstream of FcεRI activation and underscores a multi-faceted mechanism for controlling mast cell-driven allergic inflammation.

### 4.2. Upstream Regulation of LKB1: Insights into Potential Mechanisms

The upstream mechanisms regulating LKB1 activation, such as those involving Ca^2+^/calmodulin-dependent protein kinase kinase β (CaMKKβ), provide important context for understanding zingerone’s mode of action [28]. Although the present study did not directly assess the role of CaMKKβ, existing literature indicates that LKB1 can be regulated by multiple upstream kinases. Interestingly, zingerone significantly inhibited FcεRI-mediated intracellular Ca^2+^ influx (Figure 2h), suggesting that its modulation of calcium signaling could indirectly influence CaMKKβ activity—a Ca^2+^-sensitive activator of AMPK in certain contexts. Additionally, other regulators such as Akt can phosphorylate LKB1 and lead to its nuclear retention and functional attenuation [29]. The suppression of Akt phosphorylation by zingerone (Figure 2a,b) implies a potential secondary mechanism whereby zingerone enhances LKB1 activity through relief of Akt-mediated inhibition. These interconnected pathways highlight the complexity of LKB1 regulation and present meaningful directions for further research to clarify zingerone’s primary molecular target(s).

### 4.3. Distinct Mechanism and Therapeutic Positioning Among Ginger Components

When compared to other bioactive compounds in ginger, zingerone exhibits a distinct mechanism of action. For instance, 6-shogaol, another ginger-derived compound, has been reported to inhibit mast cell degranulation primarily through PPARγ activation. In contrast, the anti-allergic properties of zingerone are mediated through the LKB1/AMPK pathway. This mechanistic difference suggests that zingerone may offer therapeutic utility in cases where responses to other pathways are diminished or where a multi-targeted immunometabolic approach is advantageous. The specific targeting of the LKB1/AMPK axis by zingerone underscores its unique value as a natural product candidate for allergic conditions.

### 4.4. Therapeutic Niche and Clinical Translation

In light of the high affinity between IgE and FcεRI, biologic strategies aimed at disrupting pre-formed IgE-FcεRI complexes—such as omalizumab—represent a potent therapeutic approach [30]. Zingerone is not intended as a direct competitor to these agents but rather as a multifaceted natural compound that targets intracellular signaling cascades downstream of FcεRI aggregation. Our data indicate that zingerone effectively suppresses mast cell activation even after IgE/antigen challenge, positioning it as a suppressive agent capable of mitigating allergic responses post-exposure. This supports its potential use in the long-term management of chronic allergic inflammation, such as in mild-to-moderate asthma or atopic dermatitis, where a safe, orally administered agent is highly desirable. Furthermore, zingerone’s documented antioxidant and anti-inflammatory properties may add therapeutic value by alleviating tissue inflammation and oxidative stress associated with chronic allergic diseases [31].

### 4.5. Pharmacokinetic Considerations and Future Directions

The relatively low oral bioavailability of zingerone remains a challenge for its clinical translation [32]. Nonetheless, in vivo studies demonstrated significant efficacy at oral doses of 25–50 mg/kg, suggesting that even limited systemic exposure may suffice for biological activity, potentially due to active metabolites or high target affinity. Future work should focus on delivery optimization strategies—such as nanoparticle or liposome formulations—to improve bioavailability and therapeutic index. Although this study delineates the critical role of the LKB1/AMPK pathway, the precise upstream mechanism by which zingerone enhances LKB1 phosphorylation remains an open question. Further research using CaMKKβ-specific inhibitors (e.g., STO-609) [33] or siRNA knockdown approaches would help clarify whether zingerone directly influences CaMKKβ or operates through alternative pathways to activate LKB1.

## 5. Conclusions

In summary, this study establishes zingerone as an effective inhibitor of IgE-mediated mast cell activation and anaphylaxis, functioning through activation of the LKB1/AMPK pathway. Its unique mechanism, which differs from other ginger-derived compounds, along with its favorable safety profile, supports the potential of zingerone as a promising natural product for the management of allergic diseases. While challenges regarding pharmacokinetics remain, these findings provide a strong rationale for further pre-clinical and clinical development of zingerone and its analogs as novel anti-allergic therapeutics.

## Data Availability

The original contributions presented in this study are included in the article/Supplementary Materials. Further inquiries can be directed to the corresponding author(s).

## Supplementary Materials

The supporting information can be downloaded at: https://www.mdpi.com/article/10.3390/cimb47110963/s1.
