# Supplementary material for: Zingerone Targets LKB1/AMPK to Block FcεRI-Dependent Mast Cell Degranulation and Anaphylaxis
Source: Curr Issues Mol Biol. 2025 Nov 19;47(11):963. doi: 10.3390/cimb47110963 (PMC12650989; doi:10.3390/cimb47110963)
Supplement: Supplementary file 1 [file cimb-47-00963-s001.zip › cimb-3965197-supplementary.pdf]

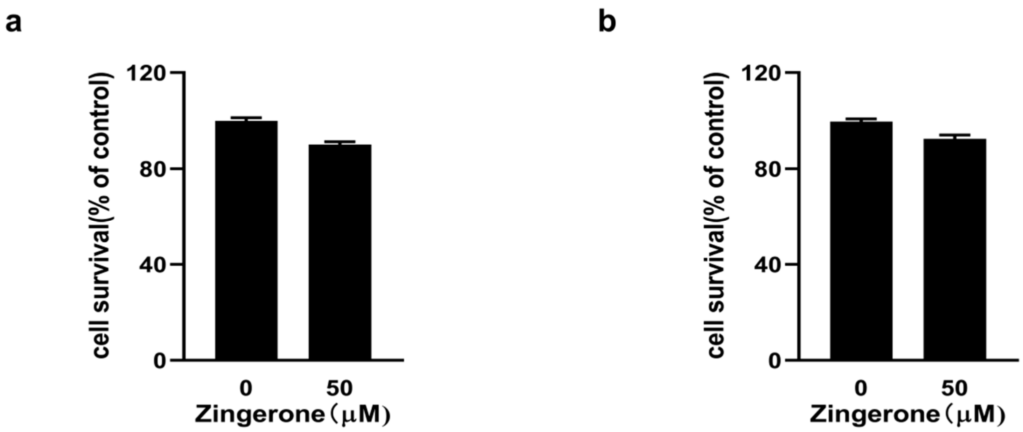

Supplementary Figure S1. Effects of Zingerone on cell viability after prolonged exposure. (a) Bone marrow-derived mast cells (BMMCs) and (b) RBL-2H3 cells were treated with 50 μM Zingerone or vehicle control (DMSO) for 24 hours. Cell viability was assessed by CCK-8 assay. Data are presented as mean ± SEM (Standard Error of the Mean) from three independent experiments (n=3).
